# NIRF-Molecular Imaging with Synovial Macrophages-Targeting Vsig4 Nanobody for Disease Monitoring in a Mouse Model of Arthritis

**DOI:** 10.3390/ijms20133347

**Published:** 2019-07-08

**Authors:** Fang Zheng, Siyu Luo, Zhenlin Ouyang, Jinhong Zhou, Huanye Mo, Steve Schoonooghe, Serge Muyldermans, Patrick De Baetselier, Geert Raes, Yurong Wen

**Affiliations:** 1 Department of Biochemistry and Molecular Biology, Key Laboratory of Environment and Genes Related to Diseases, Health Science Center, Xi’an Jiaotong University, Xi’an 710049, China; 2 The Key Laboratory of Biomedical Information Engineering of Ministry of Education, School of Life Science and Technology, Xi’an Jiaotong University, Xi’an 710049, China; 3 Research group of Cellular and Molecular Immunology, Vrije Universiteit Brussel, B-1050 Brussels, Belgium; 4 Myeloid Cell Immunology Lab, VIB Center for Inflammation Research, B-1050 Brussels, Belgium

**Keywords:** V-set and Ig domain-containing 4 (Vsig4), synovial macrophage, near- infrared fluorescence, Nanobody, in vivo imaging

## Abstract

Nanobody against V-set and Ig domain-containing 4 (Vsig4) on tissue macrophages, such as synovial macrophages, could visualize joint inflammation in multiple experimental arthritis models via single-photon emission computed tomography imaging. Here, we further addressed the specificity and assessed the potential for arthritis monitoring using near-infrared fluorescence (NIRF) Cy7-labeled Vsig4 nanobody (Cy7-Nb119). In vivo NIRF-imaging of collagen-induced arthritis (CIA) was performed using Cy7-Nb119. Signals obtained with Cy7-Nb119 or isotope control Cy7-NbBCII10 were compared in joints of naive mice versus CIA mice. In addition, pathological microscopy and fluorescence microscopy were used to validate the arthritis development in CIA. Cy7-Nb119 accumulated in inflamed joints of CIA mice, but not the naive mice. Development of symptoms in CIA was reflected in increased joint accumulation of Cy7-Nb119, which correlated with the conventional measurements of disease. Vsig4 is co-expressed with F4/80, indicating targeting of the increasing number of synovial macrophages associated with the severity of inflammation by the Vsig4 nanobody. NIRF imaging with Cy7-Nb119 allows specific assessment of inflammation in experimental arthritis and provides complementary information to clinical scoring for quantitative, non-invasive and economical monitoring of the pathological process. Nanobody labelled with fluorescence can also be used for ex vivo validation experiments using flow cytometry and fluorescence microscopy.

## 1. Introduction

Rheumatoid arthritis (RA) is a chronic autoimmune disease characterized by a failure of spontaneous resolution of inflammation [1]. Macrophages are essential in the pathogenesis of RA. An increased number of sublining macrophages in the synovium is a sign of early onset of active rheumatic disease. The degree of synovial macrophage infiltration highly correlates with the degree of inflammatory lesion and joint erosion. The depletion of these macrophages from inflamed joints benefits the RA therapy. Research has now uncovered an unexpectedly high level of heterogeneity in macrophage origin and function, and has emphasized the role of environmental factors in their functional specialization [2]. Although some results in mouse models of arthritis have contributed to our understanding of the properties of synovial macrophages, the heterogeneous populations of immune cells in RA have not been fully characterized. Good markers to discriminate these resident macrophage populations could lead to monitoring of pro- or anti-inflammatory properties of these macrophage populations that inform future therapeutic strategies.

The anatomical imaging techniques such as X-ray or ultrasonography can provide information on bone destruction. A novel method in rheumatology is mass spectrometry imaging (MSI). MSI enables the determination of the relative abundance and spatial distribution of biomolecules in joint sample tissue sections without labeling or staining [3]. It has revealed the identity and distribution of several peptides, lipids and chemical elements in cartilage, synovium and bone from patients with rheumatic diseases. Contrast-enhanced magnetic resonance imaging (MRI), ultrasonography and molecular imaging with SPECT or PET are other methods that enable the use of molecules reflecting cellular inflammatory processes to monitor disease evolution or treatment efficacy. Probes for molecular imaging targeting activated macrophages include ^18^F-polyethyleneglycol-folate, ^111^In-anti-F4/80-A3-1 and ^99m^Tc-EC20 (etarfolatide) [4]. However, these methods feature high cost and technical barriers and it is challenging to correlate these radioactive signals with post hoc in vitro analyses, such as immunohistochemistry or gene expression profiling. The more frequently used vivo imaging method in preclinical research is near-infrared fluorescence imaging. Studies of joint samples have demonstrated that near-infrared fluorescence imaging can provide complementary information to histology and histochemistry for rheumatic disorder. New molecular probes are currently being developed for arthritis imaging such as four fluorescent probes from PerkinElmer in combination [ProSense 750 fluorescent activatable sensor technology (FAST) with Neutrophil Elastase 680 FAST and MMPSense 750 FAST with CatK 680 FAST] [5], a folate receptor-targeted near-infrared dye- OTL0038 [6], and an IR-780 iodide-loaded drug for real time monitoring of in vivo drug release [7].

Near-infrared (NIR, 650–1000 nm) fluorescence imaging provides a high contrast between target and background tissues by improving tissue penetration and minimizes tissue autofluorescence. It is non-damaging radiation and less toxic for in vivo monitoring of biologic processes. Moreover, it can be easily conjugated to antibody (mAb) or antibody figments by random labeling and site-specific methods. Yet, the intact radiolabeled mAb may feature high liver uptake and slow blood elimination. The problem can be addressed by using smaller antibody fragments, such as scFv, Fab, F(ab’)_2_ or nanobodies. Nanobodies (Nbs) are single-domain antigen-binding fragments derived from naturally occurring heavy-chain only antibodies in camelids [8]. The antigen-specific Nbs have good expression in microbial systems and beneficial biochemical properties: Good solubility, good stability in harsh conditions, high affinity and specificity for the antigen, small size and strict monomeric behavior. Nbs have better imaging pharmacokinetics because they are rapidly excreted by kidneys and constitute an ideal tool for diagnostic applications in various disease areas, including infectious, inflammatory neurodegenerative diseases and tumours [9,10]. Furthermore, NIR fluorescence (NIRF) imaging is becoming a noninvasive alternative to radionuclide imaging for joints in small animals [11,12]. NIR dye labeled Nbs were documented for tumor imaging and tumor surgery imaging, for example, by using an anti-HER2 nanobody labeled with the IRDye800CW for ovarian cancer [13,14], an epidermal growth factor receptor (EGFR)-targeting nanobody for head and neck cancer [15], and ARTC2-specific nanobody for lymphoma cells [16,17]. Nbs can also be used for in vivo near-infrared fluorescence targeting of T cells [18]. Cellular imaging of immune cells such as T cells, B cells and antigen-presenting cells has provided important information on immune homeostasis, immune responses and autoimmune diseases [19]. 

V-set and Ig domain-containing 4 (Vsig4) is a Type I transmembrane protein (399 aa) and a B7 family member [20]. Vsig4 is highly expressed in lung, placenta, synovium and has unique expression on tissue macrophages such as Kupffer cells and a pivotal function in the clearance of pathogens and autologous cells [21,22]. It was also reported to be expressed in macrophages associated with lung cancer, ovarian cancer and multiple myeloma [23,24,25]. Synovium macrophages underlie multiple stages of an arthritis immune response, including its initiation, maintenance, regulation and termination. In previous studies, we have shown that specific probes targeting Vsig4 co-stain a subset of CD86 positive subpopulations in inflamed joints of arthritis mice models and can be used for SPECT/CT molecular imaging to monitor and even predict the development of RA [26,27]. The Nb119 also specifically stains the F4/80^high^ and CD11b^intermediate^ Kupffer cells (MHCII^+^CD68^+^CD64^+^ macrophages profile) in naive mice liver. It can further monitor Kupffer cells non-invasively in an acute liver mouse model [28]. In the present study, we extend the use of nanobodies for imaging the in vivo biodistribution of resident macrophages by targeting Vsig4 for usage as a florescence tool for NIRF. We show that allophycocyanin (APC) conjugated Nanobodies raised against Vsig4 could bind on peritoneal macrophages of WT C57b/6 mice by using flow cytometry. The Nb119 labelled with NIR dyes Cyanine 7 (Cy7) specifically target the inflamed joints in mice with CIA in NIRF imaging. The in vivo biodistribution of Vsig4 was specifically focusing on synovial macrophages, which correlated to Alexa fluor647 labeled Nanobody staining of these cells by using histochemistry. Thus, Nbs represent elegant fluorescence targeting probes for NIRF imaging of specific macrophages in inflamed joints.

## 2. Results

### 2.1. In Vitro Nb119-Cy7 Experiment

**Figure 1:**
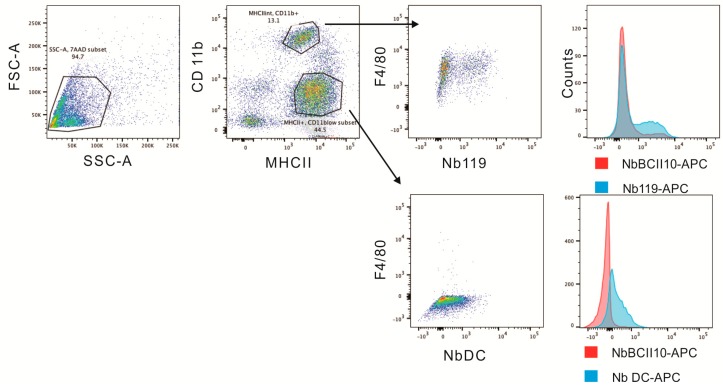
Nb119 binds to F4/80^+^ CD11b^+^ MHCII^intermediate^ peritoneal macrophages. The peritoneal cavity cells from B7 naive mice were gated according to MHCII and CD11b expression. MHCII^intermediate^ and CD11b^+^ population shows F4/80 positive and Nb119 positive. F4/80 low MHCII^high^ population is Nb119 negative. Flow cytometry histogram plots of APC-labeled Nb119 (blue) and are shown in comparison with an APC-labeled rat IgG2a kappa isotype control antibody (red).

The Vsig4-specific Nbs (Nb119) were produced in *Escherichia coli*, purified via affinity chromatography and size exclusion chromatography (SEC), as described before [29]. Flow cytometry analysis confirmed that the Nb119, but not the isotype control BCII10, recognized Vsig4 expressed on F4/80^+^ CD11b^+^ MHCII^intermediate^ peritoneal macrophages but not on F4/80^low^ MHCII^high^ peritoneal population (Figure 1). The Nb119 staining was confirmed by anti-Vsig4 antibody (Supplementary Figure S1). Flow cytometry using antibody recognizing Vsig4 specifically detects expression of the target antigen on peritoneal macrophages in naive mice.

**Figure 2:**
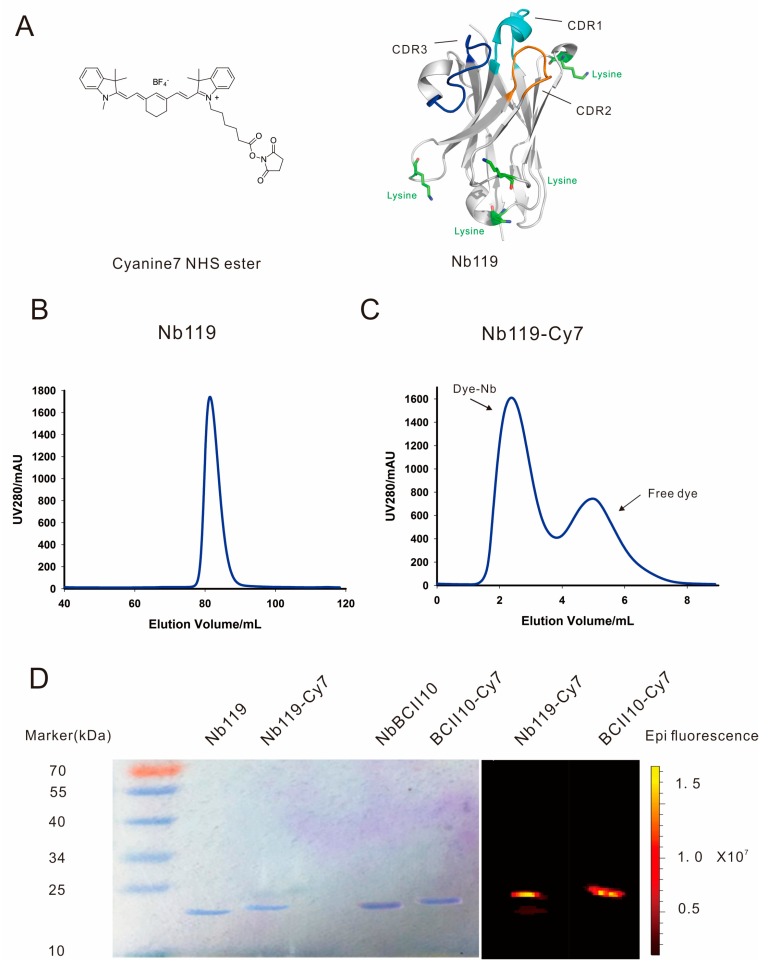
Purification and purity study of Nb119 Cy7 conjugates. (**A**) Schematic representation of Cyanine 7 NHS ester (Cy7) and Nb119 protein structure CDR1 in Cyan, CDR2 in blue and CDR3 in orange, the 4 lysines in the framework region are indicated in green. (**B**) Superdex 75 purification of Nb119. (**C**) Superdex 75 purification of Cy7 conjugated Nb119; dye-conjugated nanobody and free dye are indicated. (**D**) Coomassie-stained gel and NIRF image of unconjugated Nb119, BCII10 and respective Cy7 conjugated nanobody.

In our previous study, the crystal structure of Nb119 shows that four lysines are located in the framework but not in the antigen binding CDR regions of the nanobody and could be further labeled by Cy7 [29] (Figure 2A). NB119 was successfully purified in large yield and homogenously by Superdex 75pg column (Figure 2B) and conjugated with Cy7, followed by a desalting purification to further isolate the Cy7 labeled Nb119 from the free Cy7 (Figure 2C). The Nanobody labeling was further validated by using SDS-PAGE and NIRF imaging. A single band around 15 kDa of the purified nanobody or conjugated nanobody was noted for Coomassie stained or NIRF imaged views after SDS-PAGE. This size correlates to the predicted molecule mass of a nanobody. Conjugated NbBCII0 and Nb119 were slightly larger than the unconjugated nanobody. Conjugated NbBCII0 and Nb119 showed positive bands visualized by NIRF imaging (excitation 710 nm and emission 805 nm) which confirmed the Cy7 conjugation of the nanobody (Figure 2D).

### 2.2. NIRF-Imaging Experiments in Vivo

**Figure 3:**
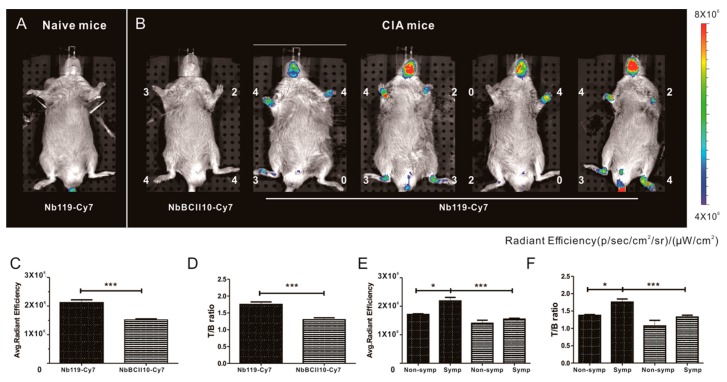
In vivo specific NIRF imaging with Cy7-labeled Nb119 tracer visualizes Vsig4 expression in arthritic joints of CIA mice. Representative NIRF image of CIA mice, 3 h after injection with Cy7 labeled Nb119 or control BCII10. (**A**) DBA/1 naive mice did not show uptake of Cy7 labeled Nb119. (**B**) Mice displaying symptoms of arthritis showed specific uptake of Cy7-labeled Nb119 in inflamed joints in correlation with clinical scores but showed no uptake of Cy7-labeled BCII10 control nanobody. Clinical scores are indicated next to each joint. Signal intensities of all injected mice are all equally leveled to allow direct and fair visual comparison. Representative images of five mice per group and at least three independent experiments are shown using National Institutes of Health color scale. NIRF imaging was performed at 3 h after injection. ROIs were drawn around arthritic joints and T/B ratios using NIRF imaging in the absence of potentially confounding signals from hind limb for semi-quantitative analyses. (**C**) Radiant efficiencies and (**D**) calculated T/B ratios of Cy7-Nb119 (solid fill) and NbBCII10 (pattern fill) are shown, *n* = 5 per group. (**E**) Radiant efficiencies and (**F**) calculated T/B ratios of Cy7-Nb119 (solid fill) and NbBCII10 (pattern fill) in non-symptomatic joints (non-symp) and symptomatic joints (symp) of five mice are compared. Data are presented as mean ± SEM from at least three independent experiments for each group. Levels of statistical significance are indicated by asterisks (*= *p* < 0.05, *** = *p* < 0.001).

Vsig4 has been reported to be an interesting target for imaging the progression of arthritis and the macrophages involved [27]. We aimed to evaluate the suitability of Cy7-conjugated nanobody for in vivo imaging of arthritic joints in the collagen-induced arthritis (CIA) model of RA in DBA/1J mice. This is a commonly used experimental model of inflammatory joint arthritis caused by a T-cell dependent, antibody-mediated auto immune response directed against cartilage type II collagen. After CIA mice immunization, some mice remained asymptomatic and others are symptomatic, and the arthritic joints among mice are not often homogeneous before day 40 post immunization [30]. Therefore, we intravenously injected Cy7-labeled Nb119 and NbBCII10 into mice at a dose of 100 μg at 28 days after subcutaneous injection of collagen II in the tail of the animals. Imaging was performed 3 h after injection of the fluorochrome conjugates (Figure 3). NIRF-imaging was used to gauge the targeting of the Nb119-Cy7 to the inflamed joints of CIA mice. The uptake of Nb119-Cy7 in the arthritic paws of CIA mice is significantly higher than in the paws of naive mice. The results showed efficient and specific labeling of arthritic joints with Nb119-Cy7 but not with NbBCII10-Cy7, reflected by a higher signal in arthritic joints for Nb119-Cy7 as compared to NbBCII10-Cy7. No strong signals of Nb119-Cy7 and BCII10-Cy7 were observed in liver or kidneys, reflecting that the fluorescent signals of the systemically administered Nbs did not pass through the fur of mice. As a result, NIRF imaging of Nb119-Cy7 allows the specific measurement of Vsig4 levels in inflamed joints. When the CIA mice were scored (0–4 for severe inflammation) for arthritis severity and subsequently injected with Nb119-Cy7 or NbBCII10- Cy7, Nb119-Cy7 uptake was readily visualized in arthritic lesions from the symptomatic CIA mice and correlated with the clinical scoring (Figure 3A,B). Semi-quantitative ROI analyses confirmed a rapidly increasing radiant efficiency and target/background (T/B) ratio of arthritic joints after injection of Nb119-Cy7 at 3 h post-injection (Figure 3C,D). The T/B ratio of symptomatic arthritic joints detected with Nb119-Cy7 was significantly higher than that of non-symptomatic arthritic joints detected with Nb119-Cy7 at 3 h post-injection (Figure 3E,F).

### 2.3. Histopathology Staining of Arthritis and Fluorescence Microscopy ex Vivo

**Figure 4:**
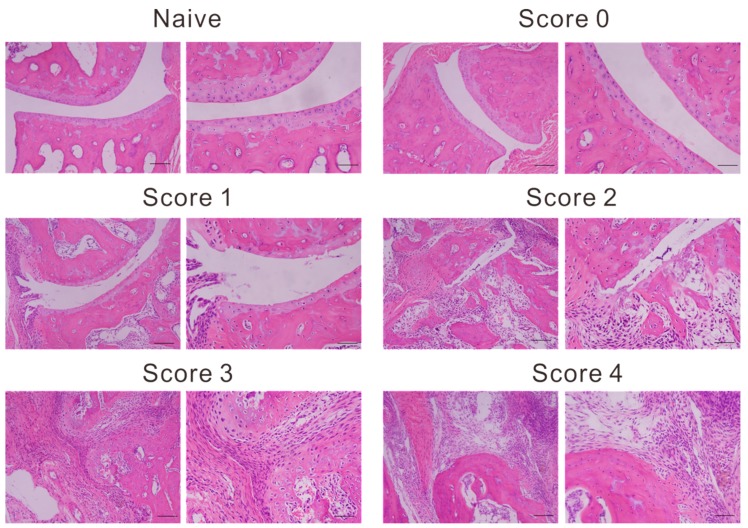
Representative images of histopathology sections. Naive and arthritic joint from each group, stained with hematoxylin and eosin (left panel magnification, ×100; right panel magnification, ×200). Photomicrographs of left hind ankles from five representative mice are shown for each clinical score group.

We further analyzed Vsig4 expression after immunofluorescence analysis. Compared with nonimmunized naive control joints, joints in CIA mice exhibited destruction typical of RA. The inflamed hind ankle sections were evaluated by histopathology staining. Hematein eosin staining revealed inflammatory cells infiltration, pannus formation, bone erosion and cartilage distraction in ankle sections with different clinical score (Figure 4).

**Figure 5:**
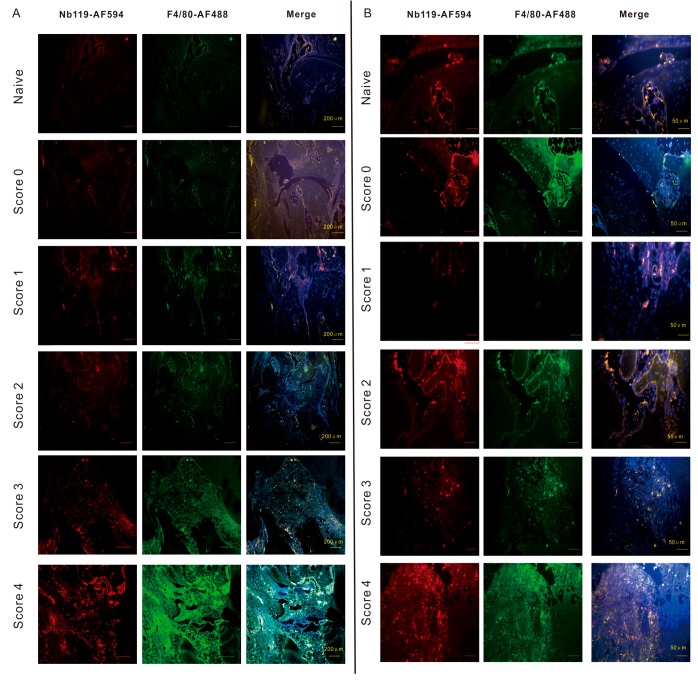
Confocal microscopy demonstrates that Vsig4^+^ F4/80^+^ macrophages increased according to the severity of arthritis. Immunofluorescence microscopy of CIA joints left hind ankles having different clinical scores. DBA-1 mice were immunized with type II collagen in complete Freund’s adjuvant. Slides were incubated with Nb119 labelled by AF549 (red) and AF488-labelled anti-F4/80 (green). Cell nuclei were stained with 4′,6-diamino-2-phenylindole (DAPI) dye (blue), colocalization of F4/80 and Vsig4 were shown in the third panel (white). (**A**) Scale bar = 200 μm. (**B**) Scale bar = 400 μm. Photomicrographs of left hind ankles from representative 5 mice are shown for each clinical score group.

Nb119- Alexa Fluor 594 was not detected in the synovial tissue sections of score 0 CIA mice. In contrast, Nb119- Alexa Fluor 594 was highly unregulated in the inflamed region lining the CIA synovium where it was co-localized with the macrophage marker, F4/80 (Figure 5).

**Figure 6:**
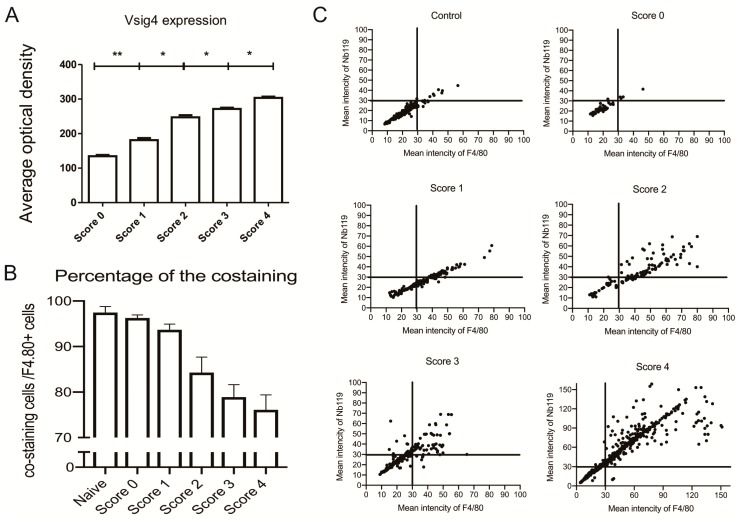
Immunofluorescence analysis in ankle sections of CIA mice having different clinical scores: (**A**) Vsig4 expression in total cells; (**B**) the percentage of Vsig4 and F4/80 contained within F4/80 positive cells. Data are presented as mean SEM. Levels of significance were calculated using one-way ANOVA. Values are means ± SEM (*= *p* < 0.05, ** = *p* < 0.01, *n* = 5). (**C**) The scatter plots of immunofluorescence analysis for Vsig4 mean intensity vs. F4/80 mean intensity in ankle sections of CIA mice having different clinical scores, scatter plots of left hind ankles from five representative mice are shown for each clinical score group.

Moreover, quantitation of Nb119 expression levels further confirmed the marked up-regulation of Nb119 associated with the clinical scoring and the severity of arthritis indicated expansion of the synovial macrophages in the late stage of arthritis (Figure 6A). The percentage of Vsig4 and F4/80 contained within F4/80 positive cells in ankle sections of CIA mice confirmed that the majority of the Vsig4^+^ cells are F4/80 positive and the Vsig4 low infiltrating macrophages increases in the late stage of arthritis (Figure 6B). Immunofluorescence analysis of the scatter plotting of each score indicated a strong correlation of the F4/80 and Vsig4 staining at the lower score group and a more heterogeneous distribution in the later stage of the inflamed joints (Figure 6C). 

## 3. Discussion

Imaging techniques will improve the understanding of the arthritis pathogenesis, assessing the course of inflammatory ailments and permitting treatment follow-up for these diseases. Examples include the labeling of antibodies or antibody fragments against markers on macrophages such as F4/80, MMR [31] and Vsig4 [27], or against the endothelial cell marker E-selectin [32]. A marker such as folate receptor, that binds folic acid with high affinity, is associated with metabolic activity in the joints and is uniquely overexpressed on activated macrophages [12,33]. Probes correlated with reactive oxygen species production, cathepsins or inflammation-associated enzymes such as MMPs or cell death were assessed in CIA by using a near-infrared PSVue 794 dye [34]. 

Fluorescence imaging has the advantages of high sensitivity and resolution and lower instrument cost compared to molecular imaging with SPECT or PET techniques, although tissue penetration represents the main challenge of optical imaging. This limitation can be partially resolved by adopting NIR light, which provides a wider dynamic range and minimal background with lower scattering than visible fluorescence detection. NIRF imaging has been frequently used in athymic nude mice. However, the DBA/1 or C6 mice, which are frequently used to establish the RA model, cause high unspecific signal originating from their fur. Since the inflamed area of RA is located in the paws, which are hardly influenced by the autofluorescence of their fur, NIRF imaging has been widely used in preclinical arthritis studies nowadays. An ideal florescence probe could be multi-functional and support different purposes such as flow cytometry, fluorescence microscopy and optical imaging. 

Nbs can be an easy and effective way to identify macrophages from the complex in vivo condition based on the detection of Vsig4 expressed on the macrophages. Recently, we developed and characterized Nb119 and evaluated Nb-based Technetium-99m radio-labelled tracers for SPECT/micro-CT and fluorine-18 labelled tracers for PET [35]. In a previous study, we confirmed that Vsig4-specific nanobody targeting synovial macrophages constitutes a specific tool for non-invasive SPECT/CT imaging as a way of detecting early signs of inflammation and assessing inflammation in multiply arthritis models in vivo [26,27]. In this study, we wanted to assess the ability of the Vsig4 nanobody to act as an optical probe for NIRF imaging. Here, we used NIRF-dye Cy7-conjugated nanobody and isotype control nanobody for a direct comparison of in vivo and ex vivo analyses. 

A limitation of our study is that we did not optimize the amount of fluorescent dyes per nanobody, which might affect the maximum achievable signal for imaging and fluorescence. Molecular random conjugation could cause further loss of affinity when lysine residues are located in or close to the antigen-binding region [13]. Recently, we crystalized the Vsig4-Nb119 complex and solved high-resolution atomic structure, showing that Nb119 has four lysines dispersed in the framework and away from the antigen-binding region [29]. Accordingly, although the labeling strategy can be improved by site-specific conjugation of the NIRF dye, Vsig4 nanobody random conjugation to primary amine groups did not affect binding performance for in vivo imaging. In the CIA model, which is readily inducible in DBA/1 mice, Nb119-Cy7 specifically detected the inflamed lesions that were found in the arthritic mice but not in the naive mice. Nb119 signals formed a positive hot spot only in inflamed paws featuring disease development in the CIA model. Radiant efficiency and T/B ratio of Cy7-Nb119 in inflamed paws was significantly higher as compared to Cy7-BCII10. Similar signals were even detected in the non-symptomatic joints group as in the symptomatic joints group by using Cy7-BCII10. 

In this study, we injected fixed doses of 100 μg nanobody regardless of the weight of the animals, instead of performing weight-adapted injections. The differences in weight of individual mice (range 20.6–24.2 g) may explain some of the observed signal variations within the Cy7-Vsig4 and Cy7-BCII10 groups. The large size of conventional antibodies impedes tissue penetration and renal elimination, resulting in suboptimal in vivo targeting. The nanobody often shows higher contrast in a short time as alternatives to monoclonal antibodies as theranostics [17,18]. Our study confirmed that Vsig4 nanobody allowed 3 h post injection imaging with high target-to-background ratio, which indicates that small single-domain nanobodies are best suited for short-term uses, such as noninvasive imaging.

These data are in line with the histochemistry in the naive and arthritic joint from each group, stained with hematoxylin and eosin corresponding to the rise of clinical scores (Figure 5). In our previous research, we showed Vsig4 expression on a subset of CD68 positive synovial macrophages [27]. CD68 is a broad marker and highly expressed by cells including circulating macrophages, osteoclasts and tissue macrophages. In the current study, we used a commonly used more specific macrophage marker F4/80 for symposium macrophages staining. The increasing signal of immunofluorescence staining with Alexa Fluor 549 labelled Nb119 and Alexa Fluor 488 labelled F4/80 confirms the massive increase of synovial macrophages in the inflamed joints. The Nb119 and F4/80 positive signals were highly correlated at the low score joints but showed less percentage of co-staining in the more serve inflamed joints, suggesting increasing heterogeneity in the population of infiltrating macrophages. Synovial macrophages are attractive targets for imaging because they seem to play a pivotal role in maintaining the chronic inflammation state in arthritis. As such, using fluorescent Vsig4 nanobody can indeed provide molecular information via flow cytometry, immunofluorescence microscopy and in vivo imaging as compared to the clinical score.

## 4. Materials and Methods

### 4.1. Mice and Cells

Eight-week old male C57Bl/6 and DBA/1 mice were purchased from Charles River Company (Wilmington, Massachusetts, USA). The experiments were approved by the local ethics committee under approval code 2015-246 on March 4th, 2015. For Figure 3, five mice per group were used for Cy7-BCII10 and Cy7-Nb119 injection. For Figure 4, Figure 5 and Figure 6, 30 left hind paws were selected from at least three individual experiments in total according to each score group (naïve, score 0–4), and 5 paws fell into each group after evaluation. The peritoneal macrophages (PECs) were obtained by injecting 8 mL of cold RPMI containing 2% endotoxin-free FCS in the peritoneum using a 10 mL syringe with an 18-gauge needle and drawing the fluid back into the syringe. PECs were washed three times by centrifugation at 300 g for 10 min at 4 °C and resuspended in PBS containing 2% FCS.

### 4.2. Induction of CIA Model and Assessment of Arthritis 

CIA was induced as described before. Briefly, 2 mg/mL chicken collagen type II (Sigma-Aldrich, Shanghai, China) in 0.1 M acetic acid was emulsified in complete Freund’s adjuvant (Difco Laboratories, Detroit, Michigan, USA). Mice were sensitized with a subcutaneous injection at the base of the tail with 100 µL emulsion. A boost injection with 2 mg/mL chicken collagen type II in incomplete Freund’s adjuvant was performed at day 21. Each limb was scored for severity of arthritis as follows: 0 = normal; 1 = redness/swelling of one joint; 2 = redness/swelling of more than one joint; 3 = swelling of entire paw; 4 = ankylosis and/or deformity. The arthritis mice at day28 post induction were used in the NIRF imaging experiments. 

### 4.3. Nanobody

Vsig4-specific Nbs (Nb119) were prepared from lymphocytes isolated from an alpaca immunized with the recombinant mouse Vsig4 protein, selected via phage display and biopanning and produced as described previously [27]. The screened Nb119 gene sequence was inserted into the pHEN6c plasmid and transfected in the *E. coli* WK6 host cell. Nbs against the β-lactamase BCII enzyme of *Bacillus cereus* (BCII10) were used as a negative control throughout the study. The pHEN6C display vectors permits the inducible periplasmic expression of Nanobodies as soluble C-terminally His6 tagged proteins in *E. coli* strain WK6. All the proteins were loaded on a NI-NTA column and further buffer exchanged and purified by size exclusion chromatography (SEC) for further usage [29].

### 4.4. Cytofluorometry Analysis

Nb119 and NbBCII10 monoclonal antibody were labelled using alexa fluor-647 lightning-link APC conjugation kit (Innova biosciences, San Diego, CA, USA). Non-activated macrophages were harvested from the peritoneal cavity of mice. About 5 × 10^5^ cells were washed three times with PBS-2% FCS and resuspended in a total volume of 100 µL. Anti-F4/80 (Cl:A3-1)/Alexa fluor 488 and Anti-Mouse CD11b (M1/70)/PE antibodies were purchased from AbD Serotec and Ebioscience. Anti-Vsig4 (NLA14) monoclonal antibody and rat IgG2a kappa isotype control were purchased from Ebioscience. Five µg of antibody or nanobody were used per 1 × 10^6^ cells for 20 min at 4 °C. Excess fluorescein labelled antibody was removed by washing with PBS-2% BSA. Stained cells were further analyzed by flow cytometry and histograms were prepared using FlowJo software (Becton Dickinson, San Jose, CA, USA).

### 4.5. Nanobody-Cy7 Labeling and NIRF Imaging in Vivo Analysis

Nbs were labelled using the Cyanine7 NHS ester (Lumiprobe, Excitation maximum, nm: 750, Emission maximum, nm: 773) according to the manufacturers’ instructions. Briefly, 2 mg nanobody was incubated overnight on ice with × 8 molar excess Cyanine7 NHS ester in 0.1 M Sodium bicarbonate solution at pH 8.5. The Nanobody-Cy7 solution was purified by size-exclusion chromatography and then passed through a Millex-GV4 0.22-mm filter (Millipore, Burlington, MA, USA). The purity of Vsig4 and BCII10 nanobody was assessed by SDS-PAGE size fractionation, followed by Coomassie brilliant blue stain. 

### 4.6. NIRF Imaging

C57BL/6J mice were injected intravenously with 100 µL of 100 µg Nanobody-Cy7. At 3 h post injection, mice were anesthetized with 3% isoflurane using an XGI-8 anesthesia system in the induction chamber; then, 1%–2% isoflurane was maintained for the duration of the imaging procedure using the isoflurane manifold housed inside the imaging chamber and the mice were positioned in the imaging chamber of the small-animal NIRF-imaging system XENOGEN IVIS^®^ SPECTRUM (Beijing, China). Nanobody-Cy7 accumulation in each paw of the mice was quantified as average radiant efficiency normalized for the size of the region of interest. Joints to background ratio was calculated by dividing the values of the joints uptake by the background value determined from the hind limb.

### 4.7. Microscopy

Inflamed joints of CIA mice were fixed in 3% paraformaldehyde (pH 7.4) and then decalcified for two weeks in 10% EDTA (pH 7.4). The joints were embedded in Tissue-Tek OCT and frozen in liquid nitrogen. Cryostat sections (5 µm) were either stained with hematoxylin and eosin as standard protocol and either incubated in 1% PBS/BR containing detection antibodies for immunofluorescence microscopy. Anti-F4/80 (Cl: A3-1)/Alexa Fluor 488 was purchased from AbD Serotec and Nb was labelled by Alexa Fluor 594 (Alexa Fluor 594 NHS Ester, Shanghai, China) purchased from Invitrogen. 2 µg of antibody and Nb in 1% PBS-BR were used per slide. Fluoro-Gel II/DAPI (Electron Microscopy Sciences, Shanghai, China) mounted slides were used for fluorescence microscopy with an UPlanFI 10×, 20× or 30× objective on an OLYMPUS BX51 microscope with OLYMPUS DP71 CCD and OLYMPUS DP Controller software (Shanghai, China).

### 4.8. Statistical Analysis

Statistical analyses were conducted using the Student’s t test and one-way ANOVA assuming unequal variances. Prism 6.0 (Graphpad software, San Diego, CA, USA) was used for statistical analyses and graph creation. *p*-values ≤ 0.05 were considered significant.

## 5. Conclusions

Our studies have confirmed that nanobody targeting Vsig4 constitute a specific tool for non-invasive fluorescence in vivo imaging as a way of assessing inflammation in arthritis models in vivo. The flow cytometry and immunofluorescence microscopy studies also indicate that fluorescence labelled NbVsig4 appears to serve as a convenient probe for distinguishing macrophages. We showed that fluorescent nanobodies are well suited as diagnostic tools for rapid and specific in vivo detection of macrophages with clear tissue penetration when performing optical imaging in vivo, providing complementary and more molecular information as compared to paw swelling and clinical scoring. As it is nonradioactive, highly sensitive, inexpensive, and comparatively easy use to produce targeted probes, we advocate the use of the NIRF imaging technique for evaluation of nanobody in preclinical molecular imaging experiments.

## Supplementary Materials

Supplementary materials can be found at https://www.mdpi.com/1422-0067/20/13/3347/s1.

## Abbreviations

NIRF	Near-infrared fluorescence
Vsig4	V-set and Ig domain-containing 4
CIA	Collagen-induced arthritis
