# Supplementary material for: NIRF-Molecular Imaging with Synovial Macrophages-Targeting Vsig4 Nanobody for Disease Monitoring in a Mouse Model of Arthritis
Source: Int J Mol Sci. 2019 Jul 8;20(13):3347. doi: 10.3390/ijms20133347 (PMC6651725; doi:10.3390/ijms20133347)
Supplement: Supplementary file 1 [file ijms-20-03347-s001.pdf]

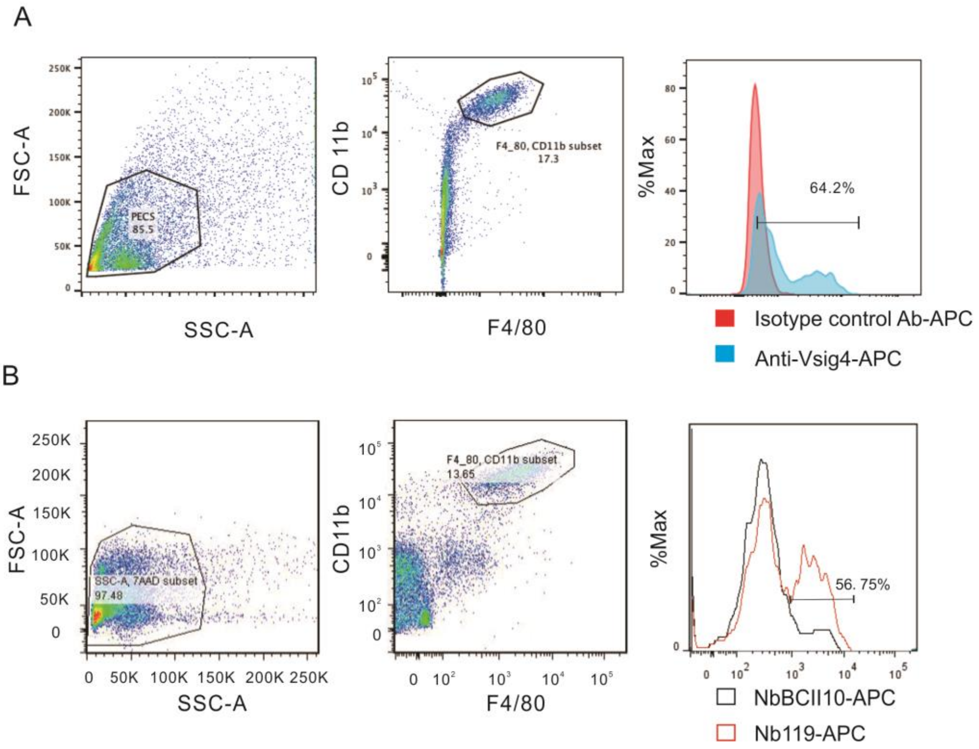

Supplementary Figure 1: Nb119 bind to Vsig4 peritoneal macrophages. A) anti-Vsig4 monoclonal antibody binds on the CD11b+ and F4.80+ peritoneal macrophages. Flow cytometry histogram plots of APC-labeled Vsig4 monoclonal antibody (blue) and staining peritoneal macrophages of naïve B6 mice are shown in comparison with a APC-labeled rat IgG2a kappa isotype control antibody (red). B) Flow cytometry histogram plots of APC-labeled Nbs (red line) staining peritoneal macrophages of naïve B6 mice are shown in comparison with a non-targeting APC-labeled NbBCII10 control Nb (black line).
